# The WHO2SAFE Score: A Predictive Tool for Postoperative Oxygen Requirement After PACU Recovery

**DOI:** 10.3390/jcm14082603

**Published:** 2025-04-10

**Authors:** Chutida Sungworawongpana, Sitthichok Chaichulee, Wongsakorn Chaochankit, Polathep Vichitkunakorn, Nachawan Gosiyaphant, Chayaporn Subanphanichkul Thongaek, Ratikorn Boonchai, Karuna Sutthibenjakul, Thadakorn Tantisarasart

**Affiliations:** 1 Department of Anesthesiology, Faculty of Medicine, Prince of Songkla University, Songkhla 90110, Thailand; chutida.s@psu.ac.th (C.S.);; 2 Department of Biological Sciences and Biological Engineering, Faculty of Medicine, Prince of Songkla University, Songkhla 90110, Thailand; 3 Division of Digital Innovation and Data Analytics (DIDA), Faculty of Medicine, Prince of Songkla University, Songkhla 90110, Thailand; 4 Department of Surgery, Faculty of Medicine, Prince of Songkla University, Songkhla 90110, Thailand; 5 Department of Family and Preventive Medicine, Faculty of Medicine, Prince of Songkla University, Songkhla 90110, Thailand

**Keywords:** oxygen inhalation therapy, risk assessment, perioperative care, respiratory insufficiency, hypoxia, general anesthesia

## Abstract

**Background:** This study aimed to develop a scoring system that predicts postoperative oxygen requirements, enhances clinical decision-making, reduces unnecessary oxygen use, and improves the efficiency of postoperative respiratory care. **Methods:** This retrospective study included patients who underwent elective non-cardiac surgery with general anesthesia between 1 January 2018 and 31 December 2022. The outcome of the study was the postoperative oxygen requirement at PACU discharge. Predictors with significance were used to create a scoring system. **Results:** Among the 42,378 cases, 14.9% required supplemental oxygen at PACU discharge. The WHO2SAFE score, which ranges from 0 to 15, incorporates eight independent risk factors, given in the mnemonic WHO_2_SAFE: intraoperative wheezing, intraoperative hypotension, obesity, operative time ≥ 180 min, sleep apnea, ASA classification ≥ 3, female, and elderly. **Conclusions:** The WHO_2_SAFE score provides a practical tool for predicting the need for supplemental oxygen at PACU discharge via the web, facilitating early intervention and efficient resource utilization. A cutoff score of 6 facilitates clinicians to identify high-risk patients who benefit from close observation while minimizing unnecessary oxygen use in low-risk individuals.

## 1. Introduction

Postoperative hypoxemia, defined as inadequate oxygen delivery to tissues, is a common and clinically significant complication among patients recovering from general anesthesia, with an incidence ranging from 12% to 50%, depending on diagnostic criteria and surgical procedures [1,2]. Persistent hypoxemia is associated with adverse outcomes, including arrhythmias, hemodynamic instability, delayed wound healing, and increased postoperative morbidity [3,4]. Its pathophysiology involves ventilation–perfusion mismatch, reduced functional residual capacity (FRC), and increased closing volume. Factors such as respiratory depression, diminished airway reflexes, and upper airway obstruction further impair oxygenation in the postoperative period [5,6,7,8].

To mitigate the risk, supplemental oxygen administration is standard practice. The British Thoracic Society (BTS) provides guidelines recommending supplemental oxygen only when clinically indicated or when peripheral oxygen saturation (SpO_2_) falls below 94%.

Despite these guidelines, oxygen is frequently prescribed excessively in the postanesthesia care unit (PACU) and continued upon discharge to the ward rather than being guided by clinical necessity. Without a systematic weaning trial, this practice results in inefficient resource utilization and missed opportunities to identify patients who are truly at risk. Moreover, the absence of predictive tools for assessing oxygen needs at PACU discharge contributes to variability in clinical practice [9].

This study aimed to develop a predictive tool for assessing oxygen requirements at PACU discharge. This tool would enable PACU teams to stratify risk, optimize postoperative monitoring, and ensure appropriate oxygen utilization. High-risk patients could receive vigilant monitoring and timely respiratory interventions, whereas low-risk patients could safely be weaned off oxygen, avoiding unnecessary therapy. Implementing this predictive score could enhance patient safety and improve resource utilization.

## 2. Materials and Methods

### 2.1. Study Design, Setting, and Ethical Declarations

This retrospective cohort study was conducted at Songklanagarind Hospital, Faculty of Medicine, Prince of Songkla University, Thailand. The study protocol was approved by the Institutional Ethics Committee (Approval No.: REC 66-392-8-1). As this study involved retrospective data analysis of anonymized medical records, the ethics committee waived the requirement for informed consent. All data management and analyses adhered strictly to institutional guidelines and ethical principles to ensure patient confidentiality and data security. The data for this study were extracted from the Hospital Information System (HIS) of Songklanagarind Hospital and the Digital Innovation and Data Analytics (DIDA) unit of the Faculty of Medicine, Prince of Songkla University. These databases contain comprehensive patient details, including demographic characteristics, surgical and anesthesia records, intraoperative events, and postoperative outcomes. Data extraction and processing were secure, ensuring accuracy, completeness, and compliance with data protection regulations.

### 2.2. Population and Characteristics

**Figure 1:**
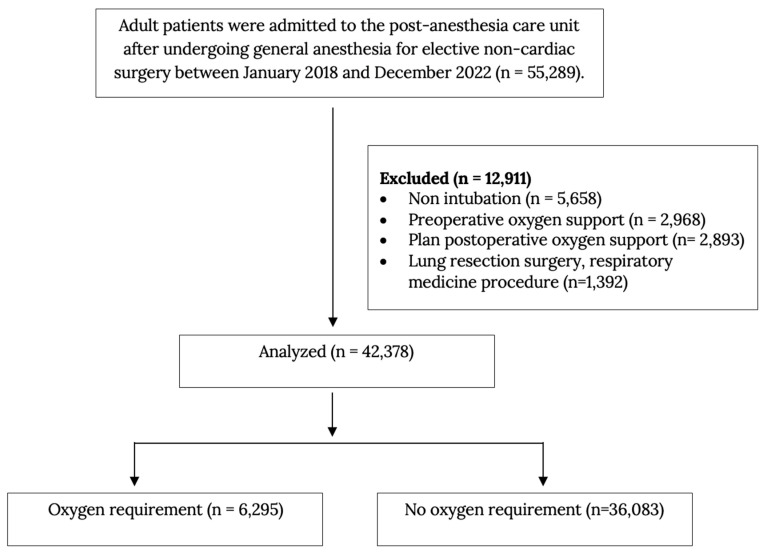
Flow diagram.

Patients 18 years and older undergoing elective non-cardiac surgery under general anesthesia who were admitted to the PACU from January 2018 to December 2022 were included. Exclusion criteria included patients with missing data (no imputation methods applied), those undergoing pulmonary surgery (e.g., thoracotomy, lobectomy, pneumonectomy), and respiratory medicine procedures (e.g., endobronchial ultrasound, bronchial stent insertion, therapeutic bronchodilation), as postoperative oxygen requirements in these cases are influenced primarily by underlying pulmonary conditions rather than general perioperative risks. Additionally, patients requiring preoperative oxygen therapy, those with planned postoperative oxygen use, and those admitted to the intensive care unit (ICU) postoperatively were excluded (Figure 1).

### 2.3. Data Variables

Demographic data, including age, sex, BMI, and smoking history, were extracted from medical records. Preoperative health status was assessed using the American Society of Anesthesiologists (ASA) classification. Underlying medical conditions, such as hypertension, ischemic heart disease, chronic obstructive pulmonary disease (COPD), asthma, and obstructive sleep apnea, were identified using hospital codes. Operative details included surgical position, surgical site, duration of surgery, estimated blood loss, and intraoperative complications such as bronchospasm (wheezing) and hypotension, as recorded in anesthetic databases. Intraoperative hypotension (IOH) was defined as a mean arterial pressure (MAP) below 60 mmHg or a reduction of 20% or more from baseline in hypertensive patients lasting over five minutes. Anesthetic variables included anesthesia technique, anesthetic agents, opioids, and neuromuscular blockers.

### 2.4. Postoperative Oxygen Requirement Measurement

Following standard practice, most patients received supplemental oxygen during transfer to the post-anesthesia care unit (PACU). Upon PACU arrival, a systematic oxygen discontinuation trial assessed each patient’s ability to maintain SpO_2_ ≥ 92% on room air until PACU discharge. Continuous SpO_2_ monitoring was performed throughout the PACU stay. If desaturation persisted despite respiratory stimulation for at least 60 s and the patient had been observed for a minimum of 30 min and met PACU discharge criteria, supplemental oxygen was administered and documented. Patients were divided into two groups based on their oxygen requirements at PACU discharge: the postoperative oxygen-requiring group and the non-oxygen-requiring group.

### 2.5. Statistical Analysis

Comparisons between patients requiring postoperative oxygen and those not were performed using the Chi-square test for categorical variables and the Mann–Whitney U test for continuous variables. Multivariable logistic regression with forward stepwise selection was used to identify independent predictors of postoperative oxygen requirement. Variables with a *p*-value less than 0.20 in the univariate analysis were included in the initial model, and the final model was refined by retaining only predictors with a *p*-value less than 0.05. To enhance clinical applicability, predictor variables were binarized. Specific cutoff values were selected based on a particular rationale. Age ≥ 60 years was set as a threshold due to the elderly increased risk of postoperative pulmonary complications [10]. A BMI ≥ 30 kg/m^2^ was set as obesity following WHO guidelines [11]. ASA class ≥ 3 indicated significant comorbidities. Operative time ≥ 180 min was defined as prolonged surgery based on the typical complexity and duration of procedures commonly performed in a university hospital setting. These cutoffs were determined based on the existing literature and clinical significance, ensuring effective risk stratification. In the multicollinearity analysis of all variables, the values were below 1.5, indicating no significant multicollinearity issue in our data. All statistical analyses were conducted using R software version 4.4.2 (R Foundation for Statistical Computing, Vienna, Austria).

### 2.6. Predictive Model Development and Internal Validation

The predictive score was developed using multivariate logistic regression. Instead of directly using the adjusted odds ratio (OR), the natural logarithm of the adjusted OR (ln [Adjusted OR]) was applied, as logistic regression inherently operates on a log-odds scale, making this transformation a more appropriate measure of association. Scores were proportionally scaled to ln(Adjusted OR) values and rounded to integers, with minor adjustments to maintain clinical interpretability and balanced weighting among predictors. To assess the internal validity of the model, both discrimination and calibration were evaluated. Discrimination performance was assessed using receiver operating characteristic (ROC) curve analysis, with the area under the curve (AUC) and its 95% confidence interval (CI) obtained through bootstrap resampling. Calibration was examined using a calibration plot by comparing predicted probabilities with observed event rates across risk, with 95% CIs generated via bootstrapping.

### 2.7. Cut-Off Selection

Sensitivity, specificity, and accuracy were calculated across a range of WHO_2_SAFE score thresholds, with corresponding 95% confidence intervals estimated using the bootstrap method. Sensitivity was defined as the proportion of patients who required oxygen and were correctly identified by the model, while specificity represented the proportion of patients who did not require oxygen and were correctly classified. Accuracy reflected the overall proportion of correct classifications. The optimal threshold was selected based on the highest F1 score, a harmonic mean of precision and recall. In this clinical context, both false positives (leading to unnecessary oxygen administration) and false negatives (resulting in failure to identify at-risk patients) carry meaningful clinical consequences. Thus, optimizing the F1 score allowed for a balanced trade-off by minimizing both types of error simultaneously.

## 3. Results

Between January 2018 and December 2022, 42,378 surgical cases were analyzed. Among these, 6295 patients (14.9%) required supplemental oxygen at PACU discharge, while 36,083 (85.1%) did not. Patients requiring postoperative oxygen were older (median age 61 years vs. 51 years), had higher BMI (25.8 vs. 23.4 kg/m^2^), and demonstrated a higher prevalence of obstructive sleep apnea (OSA) (19.3% vs. 12.2%). Hypertension was more frequently observed in the oxygen-requiring group (51.4% vs. 28%), and a higher proportion of these patients were classified as ASA ≥ III (49.2% vs. 27.3%), indicating more significant preoperative comorbidity. Intraoperatively, these patients experienced significantly higher incidences of wheezing (bronchospasm) (4.3% vs. 1.0%) and hypotension (54.1% vs. 37.9%). They also had longer operative times (median 175 min vs. 125 min) and greater estimated blood loss (Table 1 and Table 2). Multivariate logistic regression identified eight significant predictors of postoperative oxygen requirements: age ≥ 60 years, female, obesity (BMI ≥ 30 kg/m^2^), ASA classification ≥ III, OSA, intraoperative hypotension, intraoperative wheezing, and operative time ≥ 180 min (Table 3).

### 3.1. Development and Validation of WHO2SAFE Score

**Figure 2:**
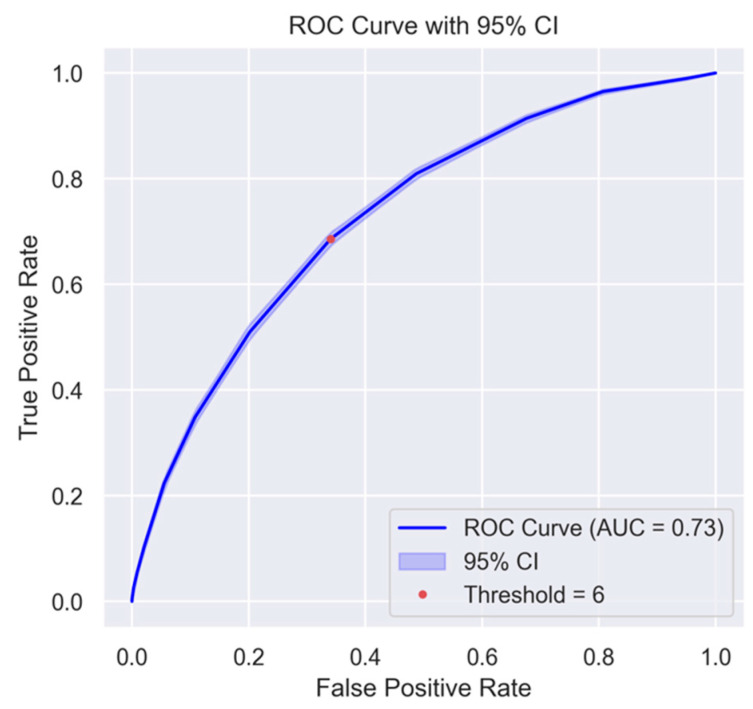
The ROC curve of the WHO_2_SAFE scoring system.

The WHO_2_SAFE score was developed using weighted values derived from multivariate logistic regression, with a total score ranging from 0 to 15. Higher scores indicate a greater likelihood of requiring supplemental oxygen at PACU discharge. Log-adjusted odds ratios were applied to standardize each predictor’s contribution, ensuring proportional weighting of risk factors while maintaining clinical practicality. The model underwent internal validation to assess its performance. It demonstrated reasonable discriminative ability, with an area under the receiver operating characteristic curve (AUC) of 0.73 (95% CI: 0.723–0.737) (Figure 2).

The area under the curve (AUC) was 0.73 (95% CI: 0.723–0.737). The shaded blue area represents the 95% confidence interval derived from bootstrap resampling. The red dot indicates the selected cutoff threshold (score = 6). Abbreviations: ROC, receiver operating characteristic.

**Figure 3:**
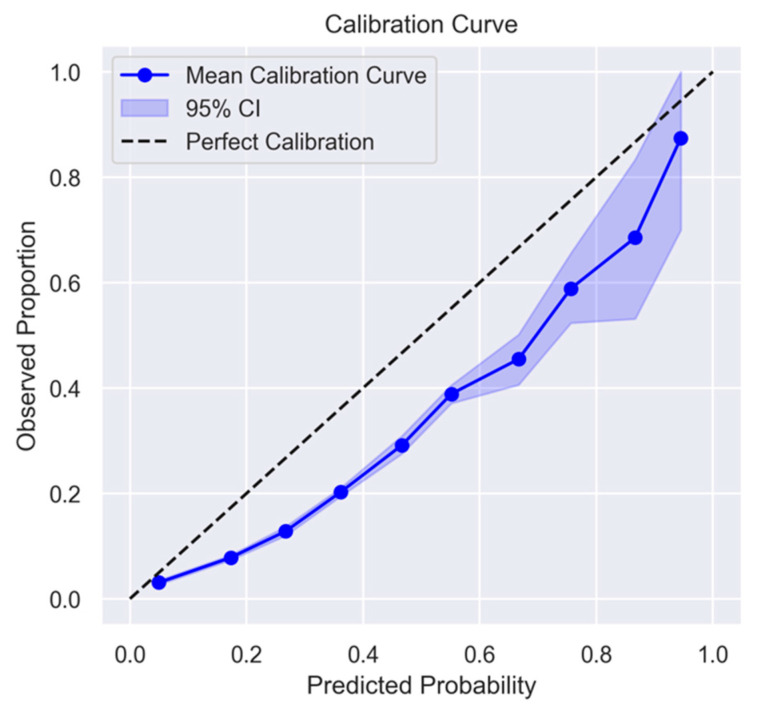
The calibration curve of the WHO_2_SAFE score. Predicted probabilities were grouped and plotted against the corresponding observed proportions. The solid blue line represents the mean calibration curve, and the shaded area denotes the 95% confidence interval obtained via bootstrap resampling. The dashed diagonal line represents perfect calibration, where predicted probabilities would exactly match observed event rates.

In addition to discrimination, the calibration plot (Figure 3) demonstrated good concordance between predicted and actual probabilities across risk. The model demonstrated good calibration for predicted probabilities below 0.8. Calibration uncertainty increased for predictions above this level, likely due to the smaller number of observations in this range. A wider confidence interval was observed at the highest decile due to fewer data points in this range.

### 3.2. Cutoff Threshold and Clinical Application

**Figure 4:**
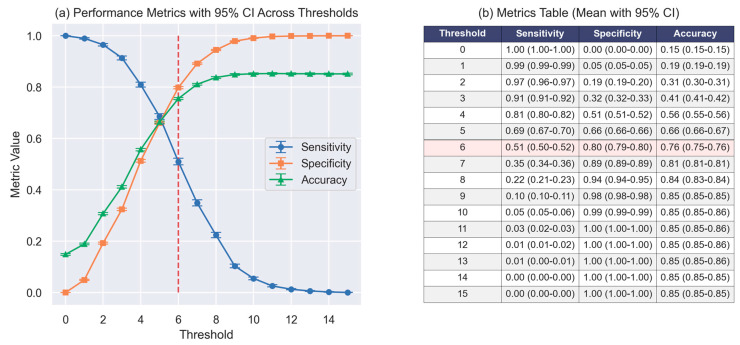
Performance metrics across different thresholds. (**a**) Sensitivity, specificity, and accuracy plotted across WHO_2_SAFE thresholds with 95% confidence intervals. The optimal threshold score of 6, indicated by the red dashed line, was selected based on balancing sensitivity and specificity. (**b**) Tables detailing the values of each metric are provided alongside the performance metric plot.

**Figure 5:**
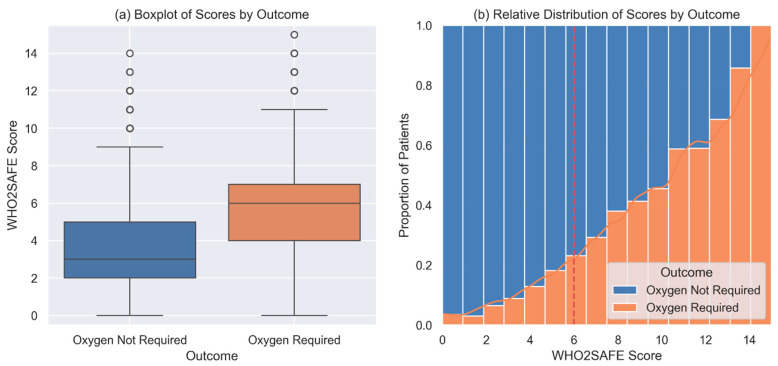
WHO_2_SAFE score distribution by postoperative oxygen requirement. (**a**) Boxplot comparing WHO_2_SAFE scores between patients who did and did not require postoperative oxygen. (**b**) Stacked bar chart showing the proportion of oxygen-requiring patients across score levels.

A cutoff score of 6 provided a balanced trade-off between sensitivity (51%), specificity (80%), and overall accuracy (76%), as illustrated in both the performance curve (Figure 4a) and the corresponding metric table (Figure 4b). The discriminative capacity of the WHO_2_SAFE score was further visualized using boxplots and stacked bar plots (Figure 5). Patients who required postoperative oxygen had higher median scores and broader interquartile ranges (Figure 5a). The distribution plot (Figure 5b) showed that the proportion of oxygen-requiring patients increased progressively with higher WHO_2_SAFE scores. A web-based calculator has been developed for easy application in clinical settings and is available at https://app.calconic.com/public/calculator/67bddafeef41c8002aebf33b?layouts=true (accessed on 9 March 2025).

## 4. Discussion

In this study, 14.9% of patients required supplemental oxygen at PACU discharge, placing our findings within the lower range of previously reported postoperative hypoxemia incidence (12–50%). However, this rate is nearly double that reported in a recent study, likely due to our cohort’s higher proportion of high-risk patients, as indicated by a threefold higher prevalence of ASA ≥ III classification [9]. Although previous studies have extensively evaluated risk factors associated with oxygen requirements during the early recovery phase following general anesthesia [12,13], the specific need for continued oxygen supplementation upon PACU discharge to the ward remains underexplored. The absence of assessment tools leads to reliance on subjective clinical judgment, potentially resulting in unnecessary oxygen overuse or delays in essential intervention [9].

### 4.1. Significance of Predictive Factors

Advanced age is a significant risk factor for postoperative hypoxia. Previous studies have associated older age with decreased lung elasticity, impaired gas exchange, and increased vulnerability to small airway collapse [10,14]. A meta-analysis identified this condition as associated with small vessel disease, which decreased vascular reactivity and inadequate tissue perfusion [15,16,17,18]. Additionally, age-related alterations in the renin-angiotensin system have been implicated in exacerbating systemic inflammation and lung injury [19]. While oxygen therapy is essential in preventing hypoxia, excessive oxygen administration may induce systemic vasoconstriction and reduce cardiac output, potentially complicating the recovery process [20]. Obesity is a significant risk factor for postoperative hypoxia primarily due to its effects on pulmonary mechanics, gas exchange, and airway function. The restrictive lung pattern in obesity is characterized by reduced expiratory reserve volume, functional residual capacity, and lung compliance [21]. Additionally, obesity is frequently associated with obstructive sleep apnea and hypoventilation syndrome, both of which further exacerbate perioperative oxygen desaturation, particularly among patients receiving opioid-based analgesia [22]. This study identifies ASA classification III as an independent risk factor for postoperative hypoxia. Similarly, Luna et al. [23] found that higher ASA classifications correlate significantly with postoperative hypoxia. Higher ASA classifications generally reflect diminished physiological reserves, impaired pulmonary function, and a reduced ability to cope with surgical stress, potentially delaying effective recovery. Additionally, intraoperative hypotension (IOH) and intraoperative wheezing have rarely been explored as potential factors intraoperatively to postoperative hypoxia in previous studies [9,18]. Although, IOH is well-documented to be associated with postoperative complications, including myocardial injury and acute kidney injury [24]. However, its relationship with increased postoperative oxygen consumption remains unexplored. IOH may result in decreased organ perfusion and tissue hypoxia, possibly heightening the need for postoperative oxygen therapy. Moreover, this study found that intraoperative wheezing is a highly significant factor. This may be explained by wheezing, often reflecting underlying hyperreactivity, leading to increased airway resistance, ventilation–perfusion mismatch, and impaired gas exchange [25]. During anesthesia, airway irritation from intubation, bronchospasm, or fluid overload can exacerbate airway obstruction and increase the risk of postoperative hypoxia [26]. These mechanisms likely contribute to the strong association in our findings. Further investigation into the impact of IOH and bronchospasm in postoperative oxygenation could provide valuable insights to enhance perioperative care. Sleep apnea screening is also a strong indicator of upper airway obstruction and reduced pharyngeal muscle tone, contributing to hypoxia [27]. Anesthesia, muscle relaxation, supine positioning, and reduced functional residual capacity (FRC) contribute to alveolar collapse. Previous studies have demonstrated that postoperative atelectasis occurs more frequently in patients with a history of snoring [28]. Therefore, preoperative identification of sleep apnea or habitual snoring is important for recognizing patients at risk of oxygen desaturation. This study defined prolonged operative time as ≥180 min, consistent with the complexity of cases in the university hospital setting. Prolonged operative time increases the risk of postoperative hypoxemia due to prolonged mechanical ventilation, increased fluid shifts, greater opioid use, and prolonged anesthetic exposure, all of which impair postoperative pulmonary function and oxygenation [29]. Utilizing lung-protective ventilation strategies and opioid-sparing multimodal analgesia reduces risk during prolonged surgical procedures [9,18]. While previous studies have identified the male sex as a risk factor for hypoxemia [9], our study found that the female sex was independently associated with increased postoperative oxygen requirement. The recent literature suggests that sex differences significantly influence respiratory physiology and postoperative outcomes. Estrogen, a primary female sex hormone, has been demonstrated to influence airway surface liquid dynamics and mucociliary clearance, potentially exacerbating conditions such as airway inflammation and hyperresponsiveness, particularly observed in females post-puberty [30]. Animal studies further indicate that estrogen may modulate lung function and airway responsiveness through effects on cholinergic pathways and airway smooth muscle, influencing susceptibility to postoperative respiratory complications [31]. These sex-specific physiological differences provide possible explanations for our findings associating female sex with increased postoperative oxygen requirements. Further research into estrogen’s role in respiratory mechanics and postoperative recovery is warranted to improve perioperative management strategies for female patients.

### 4.2. Clinical Implications and Practical Use

**Figure 6:**
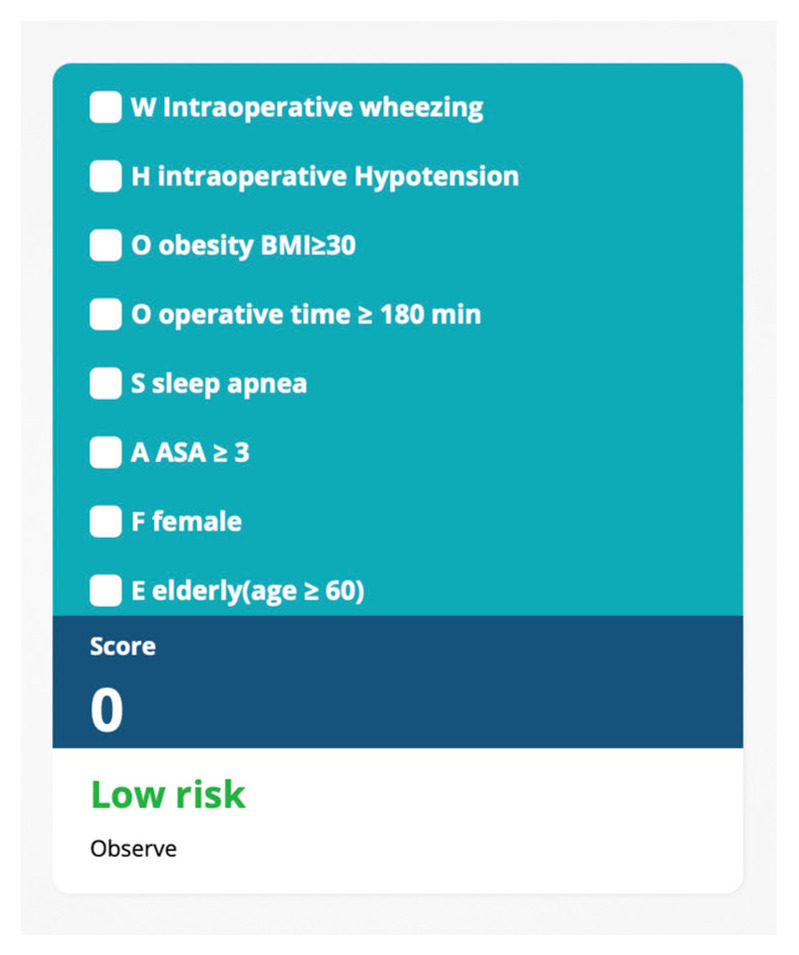
QR code of web-based application program for individual risk calculation of postoperative oxygen requirement after PACU recovery.

This study has several strengths. It utilizes a robust electronic database, ensuring data reliability, and introduces a practical scoring system accessible via a mobile-friendly web-based calculator. The tool, available at https://app.calconic.com/public/calculator/67bddafeef41c8002aebf33b?layouts=true, allows clinicians to input key patient characteristics and receive a calculated risk score with interpretation (Figure 6). It is mobile-friendly, requires no login, and is designed for convenient use at the point of care. However, the study also has limitations. As a single-center, retrospective analysis, its generalizability may be constrained. Therefore, external validation across diverse populations and clinical environments is crucial to confirm its accuracy and broader applicability before widespread clinical implementation. Clinical use should be mindful of potential risks, such as false positives, which may lead to unnecessary oxygen therapy and increased resource utilization, while false negatives could delay hypoxemia recognition, potentially increasing the risk of postoperative complications. Although the WHO_2_SAFE score is simple and easily accessible, practical implementation may encounter challenges, including variability in data availability, workflow differences, and reliance on clinical judgment. Effective integration into clinical practice will target staff training and institutional preparedness.

Future directions include formal economic impact analyses to quantify potential benefits related to oxygen resource use, hospital costs, and length of stay. Furthermore, integrating the WHO_2_SAFE score into electronic medical records (EMRs) could enable automated calculation, real-time risk stratification, and targeted postoperative interventions. This integration would enhance clinical decision-making and improve PACU efficiency.

In conclusion, the WHO_2_SAFE score is a simple and practical tool designed to predict postoperative oxygen requirements in patients undergoing general anesthesia. To enhance accessibility, we developed a mobile-friendly, web-based application that enables real-time use at the bedside. This tool supports proactive risk assessment and raises awareness of postoperative oxygen needs, facilitating timely intervention and optimizing patient outcomes.

## Figures and Tables

**Table 1:** Preoperative baseline characteristics (*n* = 42,378).

Variables	Oxygen Requirement (*n* = 6295)	No Oxygen Requirement (*n* = 36,083)	*p* Value
Age (Median [IQR])	61 (50, 71)	51 (38, 64)	<0.001
Female, *n* (%)	3777 (60)	216,721 (60)	0.938
Smoking, *n* (%)	704 (11.2)	3987 (11)	0.771
Weight, kg (Median [IQR])	64.8 (55.76, 76.2)	60 (52, 69)	<0.001
Height, cm (Median [IQR])	158 (152, 165)	160 (154, 165)	<0.001
BMI, kg/m^2^ (Median [IQR])	25.8 (22.1, 30.2)	23.4 (20.7, 26.6)	<0.001
Arrhythmia, *n* (%)	581 (9.2)	2160 (6)	<0.001
Congenital heart disease, *n* (%)	17 (0.3)	78 (0.2)	0.490
Hypertension, *n* (%)	3238 (51.4)	10,099 (28)	<0.001
Ischemic heart disease, *n* (%)	254 (4)	866 (2.4)	<0.001
Altered of consciousness, *n* (%)	23 (0.4)	575 (1.6)	<0.001
History of pulmonary TB, *n* (%)	141 (2.2)	721 (2)	0.228
Asthma, *n* (%)	213 (3.4)	791 (2.2)	<0.001
COPD, *n* (%)	136 (2.2)	444 (1.2)	<0.001
URI within 8 weeks, *n* (%)	43 (0.7)	161 (0.4)	0.016
History of COVID-19, *n* (%)	290 (4.6)	1659 (4.6)	1.000
Obstructive sleep apnea, *n* (%)	1216 (19.3)	4420 (12.2)	<0.001
ASA classification, *n* (%)			<0.001
1	71 (1.1)	2583 (7.2)	
2	3130 (49.7)	23,645 (65.5)	
3	370 (48.8)	9458 (26.2)	
4	24 (0.4)	397 (1.1)	

Abbreviations: BMI, body mass index; kg, kilogram; cm, centimeter; TB, tuberculosis; URI, upper respiratory tract infection; COVID, coronavirus disease; ASA, American Society of Anesthesiologists; COPD, chronic obstructive pulmonary.

**Table 2:** Intraoperative details. (*n* = 42,378).

Variables	Oxygen Requirement (*n* = 6295)	No oxygen Requirement (*n* = 36,083)	*p* Value
IOH, *n* (%)	3419 (54.1)	13,753 (37.9)	<0.001
Intraoperative wheezing, *n* (%)	269 (4.3)	364 (1)	<0.001
Difficult intubation, *n* (%)	21 (0.3)	46 (0.1)	<0.001
Type of anesthesia			0.002
GA, *n* (%)	5910 (93.9)	34,237 (94.9)	
GA combine with RA, *n* (%)	385 (6.1)	1846 (5.1)	
Position			<0.001
Jackknife, *n* (%)	4 (0.1)	41 (0.1)	
Kidney position, *n* (%)	56 (0.9)	174 (0.5)	
Lateral decubitus, *n* (%)	449 (7.1)	1248 (3.5)	
Lithotomy, *n* (%)	538 (8.5)	4647 (12.9)	
Prone, *n* (%)	302 (4.8)	1847 (5.1)	
Sitting, *n* (%)	33 (0.5)	166 (0.5)	
Supine, *n* (%)	4913 (78)	27,960 (77.5)	
Duration, min (Median [IQR])	175 (115, 260)	125 (75, 195)	<0.001
Blood loss, mL (Median [IQR])	50 (10, 200)	20 (5, 100)	<0.001
Anesthetic agent			
Sevoflurane, *n* (%)	3172 (50.4)	20,580 (57)	<0.001
Desflurane, *n* (%)	2320 (36.9)	7837 (21.7)	<0.001
TIVA, *n* (%)	803 (12.7)	7666(21.3)	<0.001
Morphine, *n* (%)	1127 (17.9)	6943 (19.2)	0.013
Fentanyl, *n* (%)	5748 (91.3)	31,450 (87.2)	<0.001
Succinylcholine, *n* (%)	1566 (24.9)	6858 (19)	<0.001
Cisatracurium, *n* (%)	5251 (83.4)	25,401 (70.4)	<0.001
Rocuronium, *n* (%)	15 (0.2)	169 (0.5)	0.014
Site of operation			<0.001
Intracranial, *n* (%)	107 (1.7)	600 (1.7)	
Intrathoracic, *n* (%)	362 (5.8)	507 (1.4)	
Open upper abdomen, *n* (%)	132 (2.1)	475 (1.3)	
Open lower abdomen, *n* (%)	1294 (20.5)	7735 (21.4)	
Laparoscopy, *n* (%)	1117 (17.7)	4191 (11.6)	
Groin, *n* (%)	222 (3.5)	3034 (8.4)	
Neck, *n* (%)	223 (3.5)	954 (2.6)	
Ear Nose Throat, *n* (%)	853 (13.6)	4592 (12.7)	
Spine, *n* (%)	232 (3.7)	957 (2.7)	
Vascular, *n* (%)	160 (2.5)	1672 (4.6)	
Extremities, *n* (%)	590 (9.4)	4078 (11.3)	
Eye, *n* (%)	294 (4.7)	2352 (6.5)	
Breast, *n* (%)	413 (6.6)	3108 (8.6)	
Endoscopy, *n* (%)	240 (3.8)	1502 (4.2)	
Cystoscopy, *n* (%)	56 (0.9)	326 (0.9)	

Abbreviations: GA, general anesthesia; RA, regional anesthesia; min, minute; mL, milliliter; TIVA, total intravenous anesthesia; IOH, intraoperative hypotension.

**Table 3:** Multivariate logistic regression analysis for postoperative oxygen requirement and weighted scores.

Mnemonic	Factor	*p* Value	Adjusted OR (95%CI)	ln (Adjusted OR)	Scores ^#^
W	Wheezing	<0.001	3.17 (2.67, 3.77)	1.15	4
H	Hypotension	<0.001	1.38 (1.30, 1.46)	0.32	1
O	Obesity (BMI ≥ 30)	<0.001	2.06 (1.94, 2.18)	0.72	2
O	Operative time ≥ 180 min	<0.001	2.10 (1.99, 2.23)	0.74	2
S	Sleep Apnea	<0.001	1.40 (1.30, 1.51)	0.34	1
A	ASA classification ≥ 3	<0.001	2.13 (2.00, 2.25)	0.76	2
F	Female	<0.001	1.20 (1.14, 1.28)	0.18	1
E	Elderly (Age ≥ 60 years)	<0.001	2.17 (2.04, 2.30)	0.77	2

^#^ Scores were calculated by multiplying the natural logarithm of the adjusted odds ratio (ln [Adjusted OR]) by a scaling factor of 0.32. The resulting values were rounded to the nearest whole number to improve ease of clinical use. Abbreviations: ASA, American Society of Anesthesiologists; BMI, body mass index; CI, confidence interval; OR, odds ratio.

## Data Availability

The datasets generated and/or analyzed during the current study are available from the corresponding author on reasonable request.

## Abbreviations

The following abbreviations are used in this manuscript:

ASA	American Society of Anesthesiologists
BMI	Body Mass Index
EMR	Electronic Medical Record
EBUS	Endobronchial Ultrasound
FRC	Functional Residual Capacity
GA	General Anesthesia
HIS	Hospital Information System
ICU	Intensive Care Unit
IQR	Interquartile Range
IOH	Intraoperative Hypotension
MAP	Mean Arterial Pressure
OR	Odds Ratio
OSA	Obstructive Sleep Apnea
PACU	Post-Anesthesia Care Unit
RA	Regional Anesthesia
SpO_2_	Peripheral Oxygen Saturation
TB	Tuberculosis
TIVA	Total Intravenous Anesthesia
URI	Upper Respiratory Tract Infection
